# Effects of methylthiouracil on the proliferation and apoptosis of rat bone marrow stromal cells

**DOI:** 10.3892/etm.2014.1612

**Published:** 2014-03-10

**Authors:** ZHONG-LU YE, XIAO-XIAO HOU, RE-LING CHEN, JIE DING, GUO-HUA ZHENG, MING-ZHEN CHEN, CHUAN TIAN

**Affiliations:** Department of Pediatrics, Affiliated Hospital and First Clinical College, Guangdong Medical College, Zhanjiang, Guangdong 524001, P.R. China

**Keywords:** methylthiouracil, bone marrow stromal cells, proliferation, apoptosis

## Abstract

The aim of the present study was to investigate the effects of methylthiouracil (MTU) on the proliferation and apoptosis of rat bone marrow stromal cells (BMSCs). Rat BMSCs were isolated, cultured *in vitro* and treated with various concentrations of MTU. Cell growth curves were determined using the Cell Counting Kit-8 method and the effect of MTU on BMSCs in a logarithmic growth phase was observed. BMSC apoptosis following MTU treatment was detected by flow cytometry. The experimental results demonstrated that the proliferation-inhibition effect was gradually enhanced with increasing MTU concentrations and the extension of treatment time. Statistically significant differences were observed between the treatment and the control groups (P<0.05). In addition, the BMSC apoptosis rate gradually increased with increasing drug concentrations and treatment time extension; statistically significant differences were observed between the treatment and the control groups (P<0.05). Therefore, the results of the present study demonstrated that MTU inhibited the proliferation of BMSCs and promoted apoptosis, indicating the cytotoxic effects of MTU on BMSCs.

## Introduction

Hematopoietic stem cells (HSCs) are an important index that reflect the function and status of hematopoiesis in the bone marrow, the growth of which requires a number of stimulation factors (1,2). It has been reported that >10 types of cell growth factors exist with various stimulation effects. Cell growth factors are part of a complex system that has several influencing factors (3–5). The secretion, release and function of cell growth factors depend on the hematopoietic microenvironment, which is important for HSC growth and development. Bone marrow stromal cells (BMSCs) are the main components of the hematopoietic microenvironment. Thus, studying the effects of BMSCs on hematopoiesis is important for investigating the hematopoietic function and microenvironment status. BMSCs constitute the main backbone of the bone marrow microenvironment (BMM) and perform a variety of functions involved with extracellular matrix secretion, cell surface adhesion, cytokine solubility and membrane connectivity. BMSCs can regulate and control their proliferation, differentiation and survival by adhering to HSCs (6,7). Whether under physiological status or stress conditions, the structural and functional integrity of BMSCs is important for maintaining stability and reconstructing hematopoietic function in organisms (8,9). A lack of BMSCs may result in the apoptosis of partially immature B lymphocytes (10).

Methylthiouracil (MTU; chemical name, 6-methyl-2-thiourea pyrimidine; Aladdin Co., Ltd., Chengdu, China) is one of the mostly commonly used medicines for the treatment of hyperthyroidism (11). Antithyroid drugs have been shown to damage and poison the circulatory system, among which myelosuppression is the most severe effect (12,13). These factors indicate that MTU affects HSCs to a varying degree. However, there are no studies investigating whether MTU affects the BMM, which HSCs depend on to survive, as well as the essential components of the BMM, which are the BMSCs. Therefore, the aim of the present study was to investigate the effects of MTU on BMSC proliferation and apoptosis.

## Materials and methods

### Animals

Specific-pathogen free Sprague-Dawley (SD) rats were provided by the Laboratory Animal Center of Guangdong Medical College (Zhanjiang, China). The rats were aged between 3 and 4 weeks, weighed 100–120 g and were selected without gender limitation. The study was conducted in strict accordance with the recommendations in the Guide for the Care and Use of Laboratory Animals of the National Institutes of Health. The animal use protocol was reviewed and approved by the Institutional Animal Care and Use Committee of Guangdong Medical College.

### Isolation and cultivation of BMSCs in vitro

SD rats weighing 100–120 g were selected for the study. Bone marrow mononuclear cells were extracted and the karyocyte concentration was adjusted to 2×10^5^ cells/ml. The samples were then inoculated in 25 cm^2^ plastic culture flasks. These cells were the primary cells and designated as the P0 generation. The P0 generation was cultivated in an incubator with conditions of 5% CO_2_ under a saturated humidity of 37°C. Following inoculation for 72 h, semiliquid exchange was conducted and the medium was changed every 2 days until 80–90% of the cells fused with each other, at which the digestion passage was able to be processed. Inoculative cell passage was then performed at a ratio of 1:2 and the cells were designated as the P1 generation. The culture fluid of the cell passage was changed every 2 or 3 days until the cells fused with each other and spread over the bottom of the flask. The aforementioned procedure was then repeated. By parity of reasoning, the next generation was considered the second BMSC generation and marked as the P2 generation. Subsequent cells following the P2 generation were all cultivated with 10% cell culture fluid.

### Identification of BMSCs

Surface antigen markers of BMSCs were detected by flow cytometry (FCM; Beckman-Coulter Co., Ltd., Beijing, China). The well-grown P3 generation was obtained and then rinsed three times with phosphate-buffered saline (PBS). Following digestion with 0.05% trypsin, the samples were centrifuged at a speed of 100 × g for 10 min. The cell suspension was then processed with PBS and the cells were counted simultaneously. Next, the cell suspension concentration was adjusted to 2×10^6^ cells/ml, equally transferred to four eppendorf tubes for 5 min and centrifuged at 100 × g. The supernatant was removed and 100 μl CD34-fluorescein isothiocyanate (FITC), CD45-FITC, CD90-FITC and rabbit IgG-FITC antibody (antibody titer, 1:50) were added to each tube. A blank control tube was prepared for comparison. Following thorough mixing, the cells were incubated for 30 min in the dark at room temperature. Finally, the samples were centrifuged at 100 × g for 5 min, the supernatant was removed and each tube was prepared for detection following resuspension with 500 μl PBS.

### MTT method

Well-grown P3 and P5 generations of BMSCs were obtained for inoculation and cultivated in an incubator under saturated humidity, 5% CO_2_ and 37°C for 1, 2, 3, 4, 5, 6, 7 or 8 days. Next, 10 μl MTT solution (5 mg/ml) was added to each well and the samples were incubated for 4 h at 37°C following mixing. The supernatant was removed and 150 μl dimethyl sulfoxide solution was added to each well. Following oscillation using a microdosis oscillator for 10 min, the optical density (OD) of each group was detected with a Danny microplate reader at a wavelength of 570 nm. The results were recorded and the BMSC growth curve was plotted with time as the x-axis and OD as the y-axis.

### Detection of BMSC proliferation

Well-grown BMSCs in a logarithmic growth phase were collected for the experimental study. The cell concentration was adjusted to 3–5×10^4^ cells/ml and the samples were inoculated in a 96-well plate with six repeated wells for each group. The celliferous nutrient solution of the negative control group was added to 100 μl cell culture fluid (10%), whereas the cells of the experimental groups were added to 90 μl cell culture fluid (10%) with 10 μl MTU solution at a concentration of 10, 20, 40, 80, 160 or 320 mmol/l. A blank control well was prepared for each group. Following inoculation, the cells were cultivated in an incubator with 5% CO_2_ at 37°C for 12, 36 or 48 h. Subsequently, 10 μl Cell Counting Kit-8 (CCK-8; Guangzhou Weijia Bio Co., Ltd., Guangzhou, China) solution was added to each well and further incubated for an additional 2 or 3 h at 37°C. The OD values of each group were detected using a Danny microplate reader at a wavelength of 450 nm. The results were recorded and the BMSC growth curve was plotted with the MTU concentration as the x-axis and OD as the y-axis.

### Detection of BMSC apoptosis

Well-grown BMSCs in a logarithmic growth phase were obtained for the experimental study. Cell concentration was adjusted to 2×10^4^ cells/ml and samples were inoculated in a 96-well culture plate with six repeated wells for each group. Following cultivation in an incubator with conditions of 5% CO_2_ and 37°C for 24 h, the culture medium was replaced with serum-free Dulbecco’s modified Eagle’s medium for continuous cultivation for 12 h and the supernatant was discarded. The negative control group (celliferous nutrient solution) was added to 2 ml cell culture fluid (10%), whereas the cells of the experimental groups (celliferous MTU culture medium in various concentrations) were added to 1.8 ml cell culture fluid (10%) with 0.2 ml MTU solution at a concentration of 10, 20, 40, 80, 160 or 320 mmol/l. Following cultivation in an incubator with 5% CO_2_ at 37°C for 48 h, the BMSCs of each group were digested and collected. Cells were rinsed twice with precooled PBS, centrifuged for 5 min at 100 × g and resuspended with 500 μl combined buffer solution. Next, ~5 μl annexin V-FITC and 10 μl propidium iodide (PI) solution were added and incubated for 5 min at room temperature in the dark. Annexin V-FITC presented as green fluorescence, while PI presented as red fluorescence.

### Statistical analysis

Experimental data were processed using SPSS 17.0 software (SPSS, Inc., Chicago, IL, USA) and are presented as the mean ± SD. Mean comparison among multiple samples was performed by one-factor analysis of variance, whereas comparisons between any two mean values of multiple samples were performed using the homogeneity test for variance. When the population variance was homoscedastic, Fisher’s least significance difference test was adopted, while in other cases, Tamhane’s T2 test was selected. P<0.05 was considered to indicate a statistically significant difference.

## Results

### Morphological observations of BMSCs

The extracorporeal whole bone marrow adherent method is an effective and practical method for isolating and cultivating BMSCs. The method is based on various morphological observational studies on BMSCs under an inverted phase contrast microscope (14,15). The inoculated BMSCs were spherical and had various volumes. The cells had transparent somas, but exhibited the appearance of cell growth process and were suspended in a culture flask with peripheral hemocyte series, including erythrocytes. BMSCs partially adhered to the flask wall after 12 h of cultivation. In the initial stage, the BMSCs had a short rod-like shape, which became short and fusiform and gradually presented with a fibroblast appearance. Rapid cell growth was observed between day 5 and 7 with typical colony formation. These cells were colony forming unit-fibroblasts. Cells in the center of the colony steadily increased between day 8 and 10, with close permutation and homogeneous morphology of spindle and polygon shapes. Between day 12 and 14, with the increase in the number of cell colonies, cells closely attached with each other and arranged in a direction along the long axis of the cell body, presenting with reticulate or vorticose shapes. The cells were due for passage when they fused to the monolayer gradually until they spread over the bottom of the flask. The cells began to adhere to the flask wall following cell passage for 1–2 h, with cell morphology ranging between round and spindle shapes. The cells spread over the bottom of the flask between day 3 and 4 and exhibited more homogeneous morphology. The next passage was then processed, at which the suspended erythrocytes and other hemocyte series were removed along with fluid exchange, consequently achieving cell purification.

**Figure 1:**
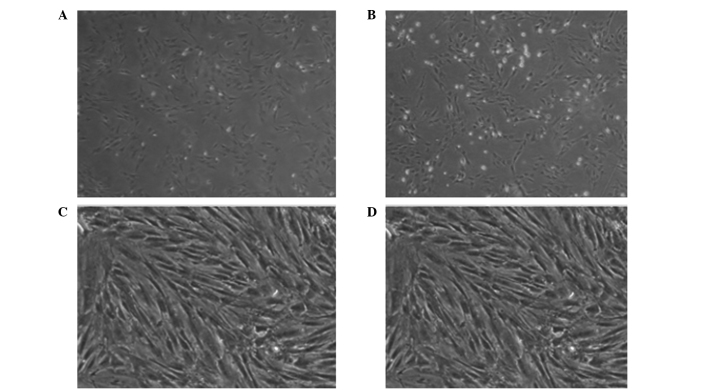
Images of BMSCs in the (A) original, (B) first, (C) third and (D) fifth generations, (magnification, ×40). BMSCs, bone marrow stromal cells.

The BMSCs were partially adhered to the flask wall after 12 h of cultivation. In the initial stage, the BMSCs had a short rod-like shape, which became short and fusiform, and gradually presented with a fibroblast appearance. Rapid cell growth was observed between day 5 and 7 with typical colony formation. These cells were colony forming unit-fibroblasts, in the center of the colony steadily increased between day 8 and 10, with close permutation and homogeneous morphology of spindle and polygon shapes. Between day 12 and 14, with the increase in the number of cell colonies, cells closely attached with each other and arranged in a direction along the long axis of the cell body, presenting with reticulate or vorticose shapes. The cells were due for passage when they fused to the monolayer gradually until they spread over the bottom of the flask (Fig. 1).

### Detection of cell surface antigens by FCM

**Figure 2:**
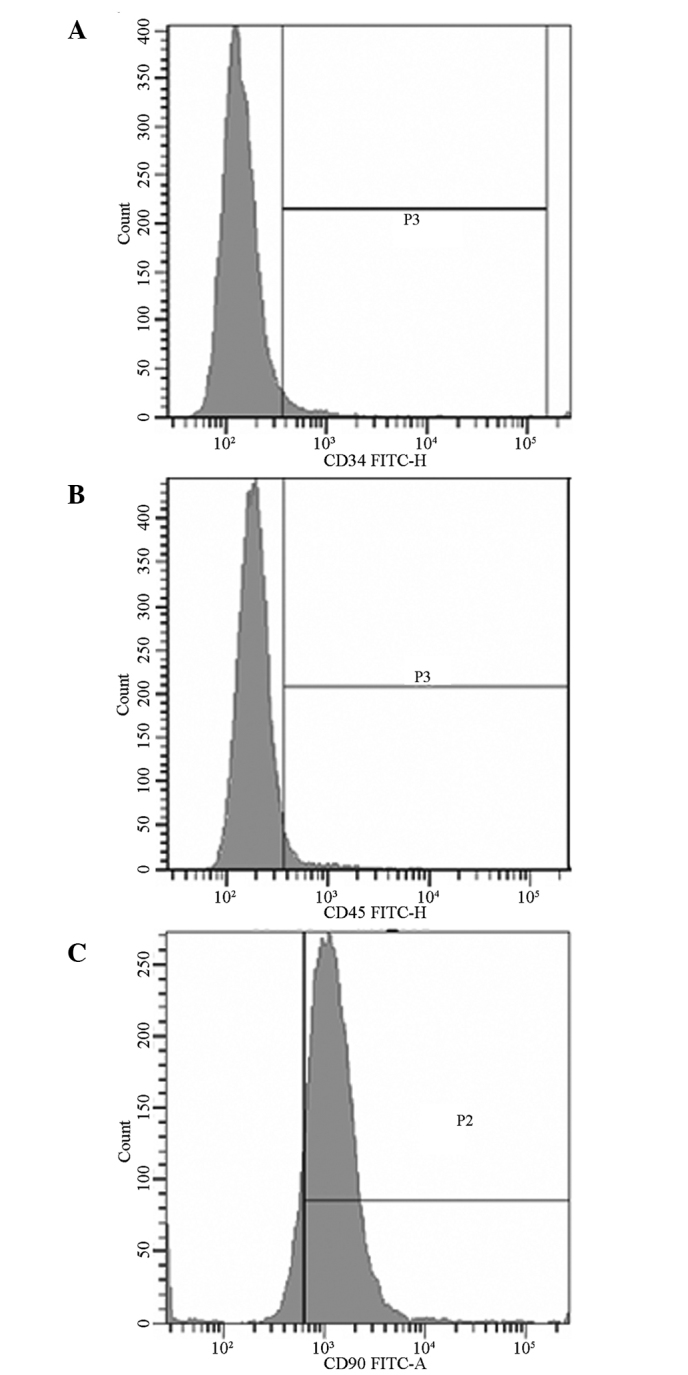
Detection of BMSC surface antigens, (A) CD34, (B) CD45 and (C) CD90, by FCM. BMSCs, bone marrow stromal cells; FCM, flow cytometry.

FCM results are shown in Fig. 2. The positive rates of CD34, CD45 and CD90 were 3.9, 5.00 and 95.60%, respectively.

### Growth curve of BMSCs

**Figure 3:**
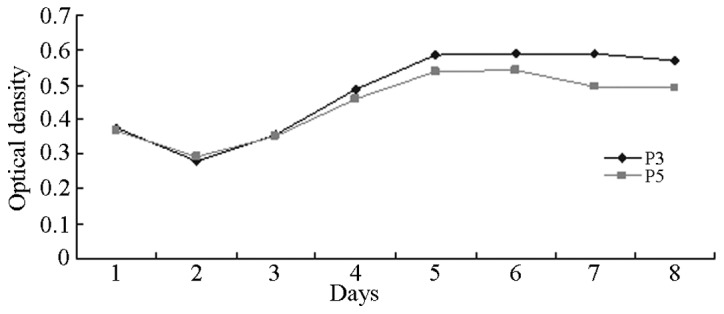
Growth curve of BMSCs. BMSCs, bone marrow stromal cells.

The proliferative growth rates of the P3 and P5 BMSC generations were observed and the results were plotted as growth curves using the MMT method. The results are shown in Fig. 3. Common features with similar sigmoid growth curves were identified. Fig. 3 demonstrates that the OD values varied slightly between day 1 and 2 following inoculation. Proliferation was not evident, whereas the OD values varied markedly between day 3 and 5, indicating that cell proliferation was more intensive than during the logarithmic growth phase. OD values started to increase from day 6 and gradually entered the platform phase. Cells in a logarithmic growth phase were selected for further experimental study.

### Effect of MTU on BMSC proliferation

**Figure 4:**
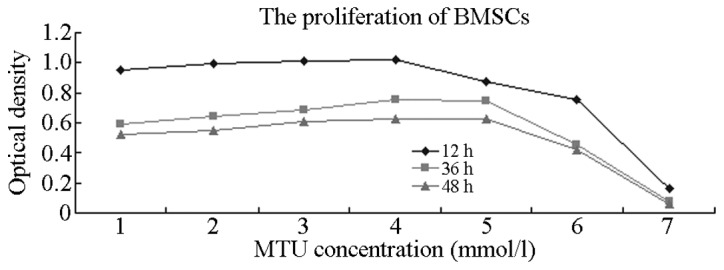
Growth curve showing the effect of MTU on the proliferation of BMSCs. 1, control; 2, 10; 3, 20; 4, 40; 5, 80; 6, 160; 7, 320 mmol/l groups. MTU, methylthiouracil; BMSCs, bone marrow stromal cells.

MTU solutions of various concentrations (10, 20, 40, 80, 160 and 320 mmol/l) were incubated with BMSCs for 12, 36 and 48 h. The effects on proliferation were detected using the CCK-8 method (Table I and Fig. 4) and the results indicated that MTU inhibited BMSC proliferation. At an MTU concentration of 40 mmol/l, the cell proliferation-inhibition rate steadily increased with increasing drug concentrations. Significant differences were identified between the control and the various treatment groups (P<0.05). The proliferation-inhibition effect was most significant when the action time was 48 h.

### Effect of MTU on BMSC apoptosis

**Figure 5:**
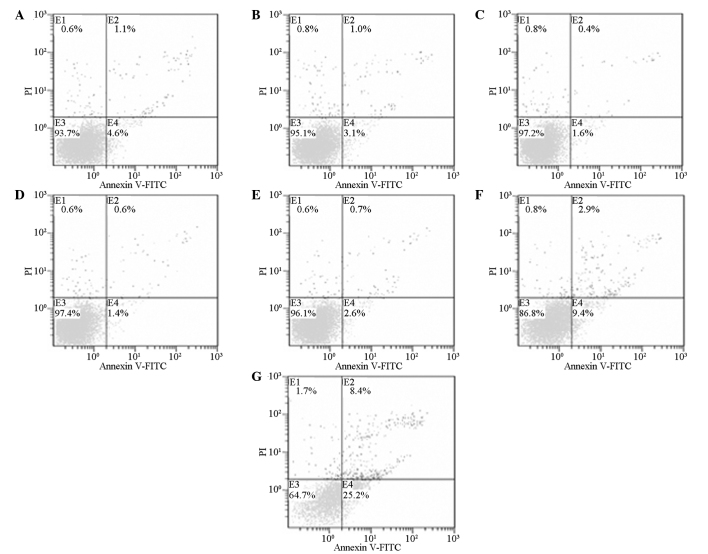
Effect of MTU on the apoptosis of BMSCs in the (A) control, (B) 10, (C) 20, (D) 40, (E) 80, (F) 160 and (G) 320 mmol/l treatment groups. E1, left superior quadrant; E2, right superior quadrant representing dead cells and apoptotic cells in an advanced stage; E3, left inferior quadrant representing normal cells; E4, lower right quadrant representing apoptotic cells at an early stage; MTU, methylthiouracil; BMSCs, bone marrow stromal cells.

Following the incubation of BMSCs with MTU solutions of various concentrations (10, 20, 40, 80, 160 and 320 mmol/l) for 48 h, the apoptosis effect was detected by FCM (Table II and Fig. 5). The results indicated that at an MTU concentration of 40 mmol/l, the cell apoptosis rate steadily increased with increasing drug concentrations. Significant differences were identified between the control and the various treatment groups (P<0.05).

## Discussion

The present study adopted a whole bone marrow adherent method to conduct *in vitro* separation and cultivation of BMSCs in SD rats. As normative surgery, the method was able to complete the separation, extraction and cultivation within a short time, thus, promoting rapid cell proliferation and passage stabilization. In addition, the method was convenient, economical, less invasive and obtained various types of matrix cell groups (16). In the present study, when the BMSCs adherently grew, the suspended HSCs and other cells were gradually eliminated with the solution replacement. Through cell passage, purified and amplified fibroblast-like cells were finally obtained with adherent growth and rapid proliferation (Fig. 1), according to the observed morphological features. FCM results indicated that the obtained cells showed positive expression for CD90, whereas negative expression was observed for CD34 and CD45. This result was in accordance with previous studies (17,18) and indicated that cultivated cells conform with the immunological marker features of BMSCs (19).

The MTT method was used in the current study (20) to detect the proliferation of P3 and P5 BMSC generations. The corresponding growth curves are shown in Fig. 3. Proliferation was solely observed during the incubation period, whereas multiple proliferations occurred during the exponential phase. This phase is the most vital period and was also the optimum phase for the current study. As the cells approached inhibition, they entered a plateau phase at which cell density saturation was reached. Cell proliferation stopped with the gradual depletion of nutrient sources in the culture solution and the accumulation of metabolites. Therefore, cells in an exponential phase were selected for empirical study.

BMSCs are the other type of stem cell present in the bone marrow, aside from HSCs. BMSCs provide indispensable support for the survival and functionalization of HSCs. Currently, various methods are available for the isolation of BMSCs, which can then be used for further study (16). However, different methods result in varying effects (18,21). The present study demonstrated that a delay in bone marrow recovery may be relevant to the injury of bone marrow stroma. A leukemic mouse model previously demonstrated that a leukemic microenvironment can inhibit the growth of normal hematopoietic progenitor cells, thus, improving and maintaining the proliferation and long-term survival of leukemic cells (22). By inhibiting the immunosuppressive function of cyclin D2, BMSCs can block leukemic cells at the G0/G1 phase of the cell cycle (23).

A CCK-8 kit was used in the current study to detect the effects of various concentrations of MTU on the proliferation-inhibition rate of BMSCs following incubation for 12, 36 and 48 h. MTU may be dissolved in ammonia solution or hydroxide alkaline solution. In the present study, 1 mol/l sodium hydroxide solution was selected to prepare the MTU solution and 10% fresh cell culture solution was diluted to the required concentration. Several concentrations of MTU were used at relatively large gradients since no previous studies on the application of MTU for *in vitro* cell experiments were identified. The CCK-8 method was performed to observe the proliferation status and to narrow the selection for a suitable medical concentration, according to the observational results. When 640 mmol/l MTU solution was added to the culture solution containing the cells, a large number of floating BMSCs were observed under an inverted phase contrast microscope. Therefore, this concentration was disregarded. The detection results of the CCK-8 kit are shown in Table I. At a final concentration of 10 or 20 mmol/l, the OD values gradually increased, but there were no statistically significant differences when compared with the control group (P>0.05). This result indicates that low concentrations of MTU have no effect on cell proliferation. At an MTU concentration of 40 mmol/l, the OD values decreased with increasing drug concentrations and this difference was statistically significant when compared with the control group (P<0.05). Comparisons among the groups that had been subjected to various action times also revealed statistically significant differences (P<0.05), indicating that cell proliferation inhibition with MTU was time-dependent. The 48 h group exhibited the lowest OD value and showed statistically significant differences (P<0.05) when compared with the 12 and 36 h groups. This result may be relevant to the chronergy of drug metabolism. Therefore, an action time of 48 h can be selected for follow-up empirical study of cell apoptosis.

An annexin V-FITC/PI double-color apoptosis kit was used to detect changes in cell apoptosis following the incubation of BMSCs with various concentrations of MTU for 48 h. As shown in Fig. 2, when compared with the control group, no statistically significant differences were observed at MTU concentrations of 10 and 20 mmol/l (P>0.05), indicating that these concentrations had no effect on the early apoptotic rate of the cells. At an MTU concentration of 40 mmol/l, the apoptotic rate increased with increasing drug concentrations; the differences were statistically significant when compared with the control group (P<0.05). Differences identified through comparisons among the various groups were statistically significant, indicating that MTU solutions at a certain concentration can dose-dependently promote the apoptosis of BMSCs.

In conclusion, MTU inhibits BMSC proliferation and promotes BMSC apoptosis. BMSCs are an important element in the BMM, indicating that MTU may cause changes in the medullary hematopoiesis microenvironment. This effect can influence the proliferation, differentiation and maturation of stem cells and their peripheral blood release or apoptosis (22,23). The promotion effect of MTU on BMSC proliferation and apoptosis also indicates that MTU may exert certain toxic effects on BMSCs (24). This observation indicates that a side effect of bone marrow suppression may result from the treatment of hyperthyroidism with MTU. However, the toxicity of MTU has provided novel ideas in the search for cytotoxic drugs.

## Figures and Tables

**Table I:** Effect of various concentrations of MTU (optical density) on the proliferation of BMSCs over 48 h (mean ± SD; n=6).

MTU, mmol/l	12 h	36 h	48 h
Control	0.948±0.100^a^	0.593±0.072^b^	0.526±0.041^b^
10	0.993±0.070^a^	0.645±0.052^a^^b^	0.552±0.038^a^^b^
20	1.014±0.047^a^	0.689±0.043^a^^b^	0.611±0.025^a^^b^
40	1.018±0.115^c^	0.757±0.067^a^^b^^c^	0.625±0.041^a^^b^^c^
80	0.877±0.052^c^	0.744±0.117^a^^b^^c^	0.623±0.073^a^^b^^c^
160	0.757±0.047^c^	0.458±0.061^c^	0.424±0.073^c^
320	0.166±0.040^c^	0.077±0.024^c^	0.058±0.032^c^

a P<0.05, vs. control group of the same time period;

b P<0.05, vs. groups with the same drug concentration but different time periods;

c P<0.05, vs. control group of the same drug concentration after 12 h.

MTU, methylthiouracil; BMSCs, bone marrow stromal cells.

**Table II:** Effect of various concentrations of MTU on the apoptosis of BMSCs over 48 h (mean ± SD; n=3).

Parameter	Apoptosis ratio, %
Control group	4.13±0.45
MTU, mmol/l	
10	2.33±0.23
20	1.67±0.12
40	1.10±0.26^a^
80	2.27±0.32^a^
160	9.50±0.66^a^^b^
320	24.2±1.40^a^^b^
F-value	513.489
P-value	0.000

a P<0.05, vs. control group;

b P<0.05, vs. different drug concentration groups.

MTU, methylthiouracil; BMSCs, bone marrow stromal cells.
